# Mortality in the first 24h of very low birth weight preterm infants in the Northeast of Brazil

**DOI:** 10.1016/j.rppede.2015.12.008

**Published:** 2016

**Authors:** Eveline Campos Monteiro de Castro, Álvaro Jorge Madeiro Leite, Ruth Guinsburg

**Affiliations:** a Escola Paulista de Medicina, Universidade Federal de São Paulo (EPM-Unifesp), São Paulo, SP, Brazil; b Universidade Federal do Ceará (UFC), Fortaleza, CE, Brazil

**Keywords:** Preterm newborn, Very low-birth weight newborn, Neonatal mortality, Early neonatal mortality

## Abstract

**Objective:**

To evaluate factors associated with neonatal death within 24 hours after birth in very low birth weight preterm newborns.

**Methods:**

Prospective cohort of live births with gestational age of 23^0/7^–31^6/7^ weeks, birth weight of 500–1499g without malformations, in 19 public maternity hospitals in nine capitals in northeastern Brazil from July to December 2007. The 19 hospitals were assessed in relation to physical resources, equipment, human resources and aiming at quality in care initiatives. Hospital, maternal and neonatal characteristics, neonatal morbidity, neonatal procedures and interventions were compared between preterm newborns that died or survived up to 24 hours of life. The variables associated with death within 24 hours after birth were determined by logistic regression.

**Results:**

Of the 627 newborns enrolled in the study, 179 (29%) died within 168 hours after birth, of which 59 (33%) up to 24 hours and 97 (54%) up to 48 hours after birth. The variables associated with death <24h were: weight <1000g (2.94; 1.32–6.53), 5th minute Apgar <7 (7.17; 3.46–14.88), male gender (2.99; 1.39–6.47). A better hospital structure was a protective factor for early neonatal death (odds ratio: 0.34; 95% confidence interval: 0.17–0.71).

**Conclusions:**

The high neonatal mortality on the first day of life in capital cities of Northeast Brazil is associated with biological variables such as weight and gender of the newborn, as well as low vitality at birth and a worse infrastructure of the hospital where the birth occurred.

## Introduction

Neonatal mortality has acquired increasing importance as the most significant cause of infant mortality. Of all neonatal deaths, 3/4 happen in the first week of life. The first day of life has the highest risk of death and accounts for 25–45% of all deaths.^1^

The neonatal mortality rate remains high in Brazil; it was of 10:1000 live births in 2011, which is 2.5 times higher than that in the United States and Canada and about 10 times higher than that of Japan in the same year.^2^ Of these deaths, in Brazil, 26% happen on the first day of life—values underestimated due to sparse data.^3^ In the Northeast region, early neonatal mortality rate related to deaths from birth up to six days old is twice higher than that of the South Region.^3^ The high number of deaths in the first week of life in Brazil, more concentrated on the first day, is related to the care provided to pregnant women and newborns during the antepartum, intrapartum, and postpartum period. Actions aimed at improving such assistance have been recommended to reduce the early neonatal deaths.^4^

In recent years, the prevalence of preterm births in Brazil is growing, first due to the increased use of assisted reproduction techniques, and second due to the quality of prenatal care and significant increase in the frequency of early terminations of pregnancy by surgical deliveries.^5^ This finding is of concern because prematurity remains one of the leading causes of death in the neonatal period and its increased frequency has nullified the improvement seen in the survival of low birth weight newborns of with improved neonatal care.^5^

In addition to the inequalities in the international and regional scenario, there are differences in neonatal mortality in different health institutions. The causes of the differences are not clear, even after adjusting for patient characteristics. Hospital care plays a key role in mortality variation found between the various centers. This fact is of importance, as the majority of deliveries in Brazil take place in health institutions.^6^ It is suggested that healthcare practices explain the differences in clinical outcome of newborns, particularly preterm neonates; however, it is difficult to identify the combination of practices considered potentially better to make an impact in reducing neonatal mortality.^7^ The identification of specific gaps in quality of care is a starting point and can support more effective interventions in reducing neonatal mortality.^8^

In this context, the aim of this study was to evaluate factors associated with neonatal death in the first 24 h of very low birth weight preterm infants born in public hospitals of capital cities of Northeast Brazil.

## Method

Hospital-based prospective cohort of live births with gestational age of 23^0/7^ to 31^6/7^ weeks and weighing ≥500 and <1500g, born in 19 public reference hospitals in the capitals of the nine Northeastern states from July to December of 2007. Patients with major congenital malformations, transferred from other institutions, and those who died in the delivery room were excluded. The study used the database of the North-Northeast Perinatal Health Network (RENOSPE), an initiative of the Ministry of Health through the Technical Department of Child Health. The project was approved by the Ethics Committee of the Maternity School Assis Chateaubriand and UNIFESP and the Data Custodian was obtained from RENOSP 2007.

The research developed by RENOSPE with data collected from the Neonatal Intensive Care Unit evaluated 36 hospitals in the Northeast states. In this study, we included only hospitals located in the capital cities (29). Of those, two were excluded for not having maternity and eight for having less than 20 patients during the collection period. Therefore, 19 hospitals were included in nine capitals of the Brazilian Northeast.

The selected hospitals were serving only patients of the Unified Health System (SUS). Data collection was performed prospectively in the chart of the newborn, from admission to discharge or death, by a field investigator trained by RENOSPE. Data collected included hospital characteristics, maternal and neonatal demographics, and clinical evolution of the evaluated newborns.

The 19 maternity hospitals were evaluated using a questionnaire filled out by health professionals with respect to physical resources, equipment, human resources, and initiatives in search of quality of care, according to the previously published methodology,^9^ and two categories were proposed: Level 1 (L1) for the 13 hospitals with better infrastructure and Level 2 (L2) for the six with less qualified infrastructure.

Data of variables related to maternal demographic characteristics, complications during pregnancy, and use of antenatal corticosteroids were collect—considered present if at least one dose of corticosteroids was given before delivery—and type of delivery. The evaluated neonatal data were related to patient characteristics, procedures in the delivery room, and the use of positive pressure ventilation (PPV) was defined when delivered with bag-mask or tracheal tube, and the presence of advanced resuscitation if PPV with heart massage and/or medications. Information concerning the newborn's temperature on admission to the ICU were collected, considering hypothermia <36 °C,^10^ respiratory distress syndrome (RDS), according to clinical and radiological criteria, and early sepsis within 72 h of life with positive blood cultures. We collect data on the procedures and interventions for neonatal care, such as the presence of transport incubator between the delivery room and ICU; transfontanellar ultrasound, if there was any examination on admission; use of continuous positive airway pressure (CPAP) at any time of admission; use of conventional mechanical ventilation at any time of admission; surfactant administration, regardless of the time used; umbilical catheterization, defined as introduction of catheter into the artery and/or umbilical vein; use of peripherally inserted central catheter (PICC) at some time of admission; assessment of pain at some point in hospitalization, defined by the application of any pain scale validated for the newborn; use of parenteral nutrition (PN), when it was used at any time of admission and if it was initiated prior to the 24 h of life. The primary outcome was death up to 24 h after birth.

Statistical analysis initially compared the variables according to the presence or absence of outcome using the Mann–Whitney or *t* test for quantitative variables and chi-square or Fisher's exact test for qualitative variables. To identify factors associated with death within 24 h after birth, logistic regression was performed with the backward stepwise method. The independent factors introduced in the initial model were those with *p*-value <0.20 in the univariate analysis and maintenance at *p*<0.05 to remain in the model. The association between the independent variables and the response variables was expressed as *Odds Ratio* (OR) and 95% confidence intervals (95%CI). The final model adjustment was evaluated using the Hosmer–Lemeshow test. Statistical analysis was performed using SPSS 17.0 software (SPSS Statistics for Windows, Version 17.0 Armonk, NY: IBM Corp.) and *p*<0.05 was considered significant.

## Results

**Figure 1:**
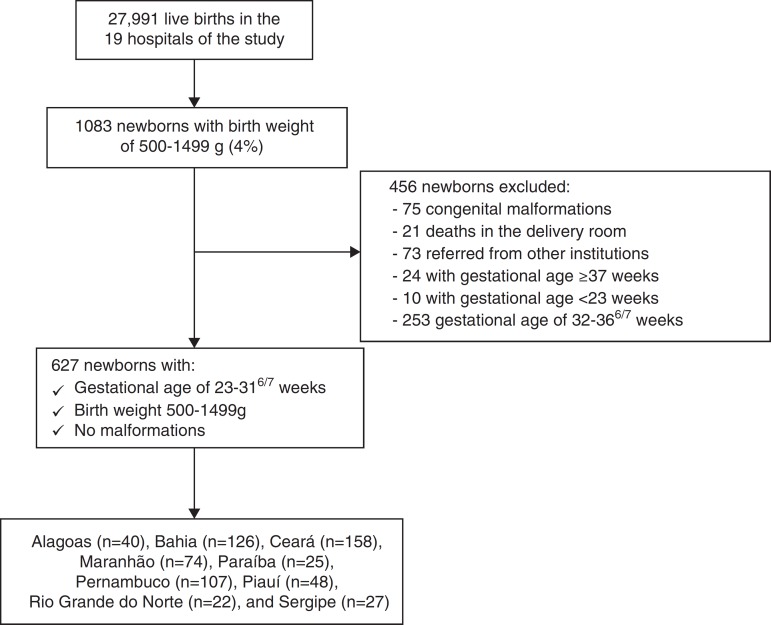
Neonates comprising the study sample based on database survey by the North-Northeast Perinatal Health Network (RENOSPE) 2007.

During the study period, 27,991 live births occurred in the 19 hospitals included in the study; of those, 1083 were newborns weighing 500–1499 g, representing 4% of total births; 456 newborns were excluded from the study and 627 preterm infants were eligible, with gestational age between 23^0/7^ and 31^6/7^, weighing between 500 and 1499 g, without congenital malformations (Fig. 1). Of neonates, 76% were born in the 13 Level 1 hospitals.

Among the 627 newborns in the study, 179 (29%) died before 168 h of life; of which, 59 (33%) within 24 h. The following distribution of deaths according to gestational age was observed: 216 infants with 23–27 weeks, of which 38 (18%) died within 24 h and 411 neonates with 28–31 weeks, of which 21 (5%) died within 24 h.

Table 1 shows the distribution of maternal characteristics according to the occurrence of newborn death up to 24 h of life. Table 2 shows the neonatal characteristics, interventions in the delivery room, and clinical complications in the first 24 h of life, according to the presence of death of the newborn in the first day of life. Table 3 shows the variables related to procedures and interventions for neonatal care, according to survival or death on the first day of life. It is noteworthy that of the 19 selected hospitals, 13 (68%) were in the category of hospitals with better infrastructure (L1 category), and in it the mortality on the first day of life of the 476 attended VLBW infants was 7%, while in the six hospitals of L2 category and in the 151 patients attended it was 17%.

**Table 1:** Maternal variables according to the presence of neonatal death 24 h after birth of very low birth weight preterm infants in the city capitals of the Northeast region.

	Death <24h (n=59)	Survival ≥24h (n=568)	*p* -value
Maternal age (years) a	24±7	25±7	0.313
Age <20 years	19 (32%)	140 (25%)	0.134
Education <8 years	32 (54%)	272 (48%)	0.214
No prenatal care	19 (35%)	88 (15%)	**0.003**
Multiple gestation	9 (15%)	78 (14%)	0.436
Diabetes in pregnancy	0	8 (2%)	0.500
Hypertensive syndrome	7 (15%)	178 (34%)	**0.004**
Peripartum infection	13 (25%)	186 (36%)	0.205
Antenatal corticosteroids	15 (27%)	275 (51%)	**0.001**
Cesarean delivery	16 (28%)	270 (48%)	**0.003**

a Variable described as mean±standard deviation.

**Table 2:** Neonatal characteristics according to the presence of death 24 h after the birth of low birth weight preterm infants in the city capitals of the Northeast region.

	Death <24h (n=59)	Survival ≥24h (n=568)	*p* -value
BW <1000g	45 (76%)	242 (43%)	**<0.001**
Birth weight (g) a	854±235	1040±252	**<0.001**
GA <28 weeks	38 (64%)	178 (31%)	**<0.001**
GA (weeks) a	27±2	28±2	**<0.001**
Male	40 (68%)	278 (49%)	**<0.001**
1-min Apgar <3	25 (45%)	57 (10%)	**<0.001**
5-min Apgar <7	34 (61%)	81 (15%)	**<0.001**
Use of PPV in DR	46 (82%)	338 (60%)	**<0.001**
Advanced resuscitation	11 (21%)	26 (5%)	**<0.001**
Temp. on admission (°C) a	35.3±0.8	35.6±0.7	**0.003**
Hypothermia on admission	41 (91%)	429 (82%)	**0.008**
RDS	51 (90%)	487 (87%)	0.360
Early sepsis BC+	2 (4%)	32 (6%)	0.436

a Variable described as mean±standard deviation; BW, birth weight; GA, gestational age; PPV, positive pressure ventilation; DR, delivery room; advanced resuscitation, PPV with heart massage and/or medications; RDS, respiratory distress syndrome; BC+, positive blood culture.

**Table 3:** Procedures and interventions for diagnostic and therapeutic neonatal care, according to the presence of neonatal death 24 h after the birth of very low birth weight preterm infants in the city capitals of the Northeast region.

	Death <24 h (n=59)	Survival ≥24 h (n=568)	*p* -value
Transport in incubator a	14 (26%)	248 (45%)	**0.004**
Surfactant use	35 (59%)	366 (64%)	0.260
Surfactant up to 2 h of life	23 (66%)	243 (66%)	0.533
CPAP	11 (19%)	423 (75%)	**<0.001**
Mechanical ventilation	51 (86%)	418 (74%)	**0.018**
Transfontanelar US	1 (2%)	316 (56%)	**<0.001**
Umbilical catheterization	43 (73%)	436 (77%)	0.300
PICC	1 (2%)	133 (23%)	**<0.001**
Use of pain scale b	2 (4%)	152 (28%)	**<0.001**
Parenteral nutrition	5 (9%)	415 (73%)	**<0.001**
PN <24h [n PN <24h/n PN (%)]	2/5 (40%)	192/420 (46%)	0.571
Hospital category (L1) c	33 (56%)	443 (78%)	**<0001**

a Transportation from delivery room to the ICU at birth; HL, hours of life; CPAP, continuous positive airway pressure by nasal prongs; US, ultrasound; PICC, peripherally inserted central venous catheter.

b Use of any validated rating scale of pain; PN, parenteral nutrition; PN24, beginning of parenteral nutrition in the first 24 h of life.

c Hospital category L1, when the hospital served more than 60% of the items described in the method.

Table 4 shows the results of multivariate analysis for death on the first day of life. It is noted that the better infrastructure-L1 hospitals-was a protective factor for the assessed outcome; birth weight <1000g, Apgar score at 5 min <7, and male behaved like risk factors. The logistic regression model was adjusted for the variables presence of prenatal care, cesarean delivery, and use of antenatal corticosteroids. The Hosmer–Lemeshow test shows the proper fit of the model, with *p*=0.712.

**Table 4:** Independent variables associated with the response variable “death within 24 h after birth of very low birth weight preterm infants in the city capitals of the Northeast region”: final model of the logistic regression analysis.

	*Odds Ratio*	Confidence interval 96%
Hospital category N1	0.34	0.17–0.71
Birth weight <1000g	2.94	1.32–6.53
5-min Apgar <7	7.17	3.46–14.88
Male	2.99	1.39–6.47

Model adjusted for the presence of prenatal care, cesarean delivery and use of antenatal corticosteroids.

## Discussion

This multicenter study shows that neonatal mortality in the first 24 h of life is high in very low birth weight preterm infants in the capital cities of northeastern Brazil, compared with the more developed regions of Brazil and developed countries. Of the 627 studied neonates, 59 (9.4%) died in the first 24 h. In the study of university public maternities in the South and Southeast performed by the Brazilian Neonatal Research Network in 2004, of 560 newborns evaluated, excluding deaths in the delivery room, 25 (4.5%) died within 24 h. It is noteworthy that patients weighing between 400 and 1499 g were included in the study, which may have contributed to increase this percentage.^11^ Mohamed et al.,^12^ in a cohort study of neonates with birth weight between 500 and 1499 g performed in the United States between 1997 and 2004, found that among the 91,578 infants studied, 4579 (5%) died within 24 h. The earlier the death of the newborn, the more it is related to the antenatal conditions, peripartum, and childbirth itself. Death on the first day after birth is associated with social and economic determinants related to the quality of maternal health care.^13^ In this line of thought, there was an attempt to understand how the factors associated with death within 24 h after birth behaved.

Among the factors associated with neonatal death on the first day of life there are three groups of variables: those related to the quality of perinatal care (hospital category), those related to neonatal biological variables (birth weight and male), and those reflecting care during labor and delivery (Apgar score at 5 min).

Regarding hospital structure, all hospitals in the study belonged to the Unified Health System (SUS) and several authors reported high rates of neonatal mortality in SUS public hospitals.^6^^,^ 13 ^,^ 14 One must remember that the socioeconomic characteristics of SUS users in itself may lead to worse survival results. However, Alleman et al.^15^ reported that the differences in neonatal mortality between the several units cannot be explained only by the diversity of characteristics of patients admitted there, but it may be more related to the use of interventions predictive of survival. In this sense, it is noted that in this study the best hospital infrastructure protected the newborn against death up to 24 h after birth, decreasing the chance of its occurrence by 66%. It is noteworthy that, in the absence of a standardized evaluation model for hospital maternities associated with the risk of neonatal death, a weighted score of the characteristics related to hospital structure was established, in which the less frequent characteristics were most valued. According to this score, it was seen that mortality on the first day of life of VLBW infants was two-fold higher in hospitals with poor infrastructure (L2), compared with those who with better conditions (L1). Among the studied characteristics, more sophisticated technological features, such as bedside ultrasound and echocardiography, and the potential to reflect on the medical practice used and learn from possible mistakes and omissions through clinical meetings were absent from most institutions classified as L2. That is, although all hospitals analyzed were public and linked to SUS, some have invested more in diagnostic tools for premature patients, technology dependent for their survival, and some have invested more in the training of human resources, fundamental to apply the technological resources in this extremely vulnerable population of newborns.

Regarding the biological factors related to neonatal death within 24 h after birth, we highlight the extremely low birth weight and male. Among the 287 infants <1000g analyzed in this study, 45 (15.7%) died within 24 h, accounting for 76% of neonates who died during this period. Birth weight <1000g increased three times the risk of death within 24 h, compared to the weight of 1000–1499g. In the American Neonatal Network, among the 6780 extreme low birth weight preterm infants born between 1998 and 2003, mortality was 14.3% in the first 24 h.^16^ In a population-based cohort in São Paulo between 2000 and 2001, of all deaths up to 12h of life, 86% had birth weight <1000g.^17^ Lee et al.^18^ proposed a model that includes both gestational age and birth weight in death risk assessment, with an increased risk for each decrease of 100g in birth weight and a week in gestational age. In the present study, in the 109 VLBW infants between 500 and 749g, mortality in the first 24 h was 19.3%, while in the 178 with 750–999g this value was 13.5%. Mohamed et al.^12^ analyzed the survival of VLBW infants between 1997 and 2004 in the United States. In the 18,863 newborns weighing between 500 and 749g, mortality rate in the first day of life was 19.6%, while in the 17.433 weighing 750–999g it was 3.1%. Probably, the lower mortality rate in the weight range of 500–749g at birth of the Northeastern maternities compared to the US reflects a large number of infants with intrauterine growth restriction and more advanced gestational age, which favors the chance of survival. Indeed, of the 109 infants weighing between 500 and 749 g, 26 (24%) had gestational age of 28–31 weeks. It is noteworthy that in the present study, among the 627 newborns with gestational age of 23 weeks to 31 weeks and six days, birth weight between 500 and 1499g, and without birth defects, 21 (3%) died in the delivery room and lost the opportunity of care in the intensive care unit.

The male gender is strongly associated with death in the first 24 h of life, with a risk of death three times higher compared to women. The same finding was seen in-hospital mortality in other studies.^19^^,^ 20 Itabashi et al.^21^ evaluated 3065 extreme low birth weight Japanese infants and found a 1.6 higher risk of hospital death in male. Australian research findings suggest that fetal growth and survival are mediated by sex-specific functions of human placenta, with better adaptation and response to oxidative stress in female.^22^

Among the factors related to care in childbirth associated with mortality within the first 24h after birth, the Apgar score <7 at 5 min is highlighted. In a population-based research in the United States performed between 2001 and 2002, Lee et al.^18^ evaluated a cohort of 690,933 infants between 24 and 36 weeks of gestation and found association between low Apgar scores at 5 min and neonatal mortality. In Sweden, between 2000 and 2002, the analysis of 156 preterm infants with 23:24 weeks gestational age also identified association between low Apgar scores at 5 min and neonatal mortality in the first 24h of life.^23^ The correlate of low vitality at birth is the need for advanced procedures of neonatal resuscitation, defined as intubation and/or heart massage and/or use of medications in the delivery room. Study of the American Neonatal Research Network, with data from 9565 preterm infants between 22 and 28 weeks, found that 67% received ventilation at birth by tracheal cannula, 8% required cardiopulmonary ressussitation, and 5% medications.^24^ National data indicate that the need for advanced resuscitation in very low birth weight preterm infants increases twice the chance of in-hospital death or survival with bronchopulmonary dysplasia and/or severe intracranial hemorrhage.^25^ In the present study, it was found a strong association of 5-min Apgar score <7 with death in the first 24 h of life. The risk was 7.2 higher than those with 5-min Apgar score=8–10. Proper care during birth of preterm infants in the reference hospitals in the Northeastern city capitals is outlined as a priority to mitigate the transition difficulties for the extra-uterine life, facilitate the cardiorespiratory adaptation, and enable clinical stability and reduced mortality in the first day of life.

It is important to emphasize that the use of secondary data has limitations and difficulties inherent in the methodology and favors failures in completing the forms. Thus, eight hospitals were excluded from the study for presenting non-representative sample of its institutions. Furthermore, the interval of seven years between data collection and its analysis shows the difficulties for consolidating information in units where there is no training for research. Nevertheless, this is the first study with prospective collection of reference maternities data in the Northeast region that helps outline a picture of the service to labor and delivery of very low birth weight preterm infants, which contribute significantly to neonatal children mortality and influence human development index of these places. In this context, the results indicate that early neonatal mortality especially in the first 24 h is high in very low birth weight preterm patients in the city capitals of the Northeast region compared to the more developed regions of Brazil and in developed countries. Our findings reveal the existence of failures in the intensive care units assessed, with poor hospital infrastructure and high contribution of extremely low birth weight and perinatal asphyxia for mortality within the first 24 h of life. Overcoming these data requires a government policy that clearly assigns responsibilities for actions aimed at improving the quality, with priority settings, selection and definitions of interventions and constant evaluation.
