# Supplementary material for: Mortality in the first 24h of very low birth weight preterm infants in the Northeast of Brazil
Source: Rev Paul Pediatr. 2016 Jan-Mar;34(1):106–13. doi: 10.1016/j.rppede.2015.12.008 (PMC4795729; doi:10.1016/j.rppede.2015.12.008)
Supplement: Translation

# Translation: Mortalidade com 24 horas de vida de recém-nascidos pré-termo de muito baixo peso da Região Nordeste do Brasil

**Authors:** Eveline Campos Monteiro de Castro, Álvaro Jorge Madeiro Leite, Ruth Guinsburg

**Affiliations:** a a Escola Paulista de Medicina, Universidade Federal de São Paulo (EPM-Unifesp), São Paulo, SP, Brasil; b Universidade Federal do Ceará (UFC), Fortaleza, CE, Brasil

**Keywords:** Recém-nascido pré-termo, Recém-nascido de muito baixo peso, Mortalidade neonatal, Mortalidade neonatal precoce

## Abstract

**Objetivo:**

Avaliar os fatores associados ao óbito neonatal até 24 horas após o nascimento de recém-nascidos pré-termo de muito baixo peso.

**Métodos:**

Coorte prospectiva dos nascidos vivos com idade gestacional de 23^0/7^-31^6/7^ semanas, peso ao nascer de 500-1.499g sem malformações em 19 maternidades públicas de nove capitais na Região Nordeste do Brasil de julho a dezembro de 2007. Os 19 hospitais foram avaliados com relação aos recursos físicos, equipamentos, recursos humanos e iniciativas de busca de qualidade no atendimento. As características hospitalares, maternas e neonatais, a morbidade neonatal, os procedimentos e as intervenções neonatais foram comparados entre os recém-nascidos pré-termo que morreram ou sobreviveram até 24 horas. As variáveis associadas ao óbito até 24 horas após o nascimento foram determinadas por regressão logística.

**Resultados:**

Dos 627 recém-nascidos incluídos no estudo, 179 (29%) morreram até 168 horas de vida, dos quais 59 (33%) até 24 horas e 97 (54%) até 48 horas. As variáveis associadas ao óbito <24h foram: peso <1.000g (2,94; 1,32-6,53), Apgar 5° minuto <7 (7,17; 3,46-14,88), sexo masculino (2,99; 1,39-6,47). A melhor estrutura hospitalar foi fator de proteção para o óbito neonatal precoce (*Odds Ratio* 0,34; Intervalo de Confiança 95% 0,17-0,71).

**Conclusões:**

A elevada mortalidade neonatal no primeiro dia de vida nas capitais do Nordeste brasileiro associa-se a variáveis biológicas, como o peso e o sexo do recém-nascido, assim como à baixa vitalidade ao nascer e à pior infraestrutura do hospital no qual o parto ocorreu.

## Introdução

A mortalidade neonatal vem assumindo importância crescente como componente mais expressivo da mortalidade infantil. De todos os óbitos neonatais, 3/4 ocorrem na primeira semana de vida. O primeiro dia de vida apresenta o maior risco de morte e representa 25-45% de todas as mortes.^1^

No Brasil, a taxa de mortalidade neonatal persiste elevada, é de 10/1.000 nascidos vivos em 2011, 2,5 vezes superior à dos Estados Unidos e do Canadá e cerca de dez vezes maior do que a do Japão no mesmo ano.^2^ Dessas mortes, no Brasil, 26% acontecem no primeiro dia de vida, valores esses subestimados em decorrência da pouca notificação.^3^ Na Região Nordeste, a taxa de mortalidade neonatal precoce, referente aos óbitos ocorridos entre o nascimento até seis dias de vida, é duas vezes mais elevada do que a da Região Sul.^3^ O elevado número de óbitos na primeira semana de vida no Brasil, mais concentrado no primeiro dia, tem relação com a assistência prestada à gestante e ao recém-nascido durante os períodos pré-parto, intraparto e pós-parto. Ações dirigidas à melhoria dessa assistência têm sido recomendadas para reduzir as mortes neonatais precoces.^4^

A prevalência da prematuridade vem crescendo nos últimos anos no Brasil, decorrente, por um lado, da disseminação de técnicas de reprodução assistida e, de outro, da qualidade da assistência no pré-natal e do aumento significativo da frequência de interrupções antecipadas da gravidez por partos cirúrgicos.^5^ Essa constatação constitui motivo de preocupação, uma vez que a prematuridade persiste como uma das principais causas de óbito no período neonatal e a sua frequência aumentada tem anulado os avanços conseguidos na sobrevida de recém-nascidos de baixo peso com a melhoria da atenção neonatal.^5^

Além de desigualdades no cenário internacional e regional, existem diferenças na mortalidade neonatal em diferentes instituições de saúde. Não são claras as causas dessas diferenças, mesmo após ajuste para diversas características dos pacientes. Os cuidados hospitalares desempenham papel fundamental na variação de mortalidade encontrada entre os diversos centros. Tal fato se reveste de importância, uma vez que a maioria dos partos no Brasil ocorre em instituições de saúde.^6^ Sugere-se que as práticas assistenciais expliquem as diferenças encontradas no desfecho clínico dos recém-nascidos e, especialmente, dos neonatos pré-termo; no entanto, é difícil identificar a combinação de práticas consideradas potencialmente melhores para promover impacto na redução da mortalidade neonatal.^7^ A identificação de falhas específicas na qualidade de atendimento constitui ponto de partida e pode subsidiar intervenções mais efetivas na redução da mortalidade neonatal.^8^

Nesse contexto, a presente pesquisa tem por objetivo avaliar os fatores associados ao óbito neonatal até 24 horas após o nascimento de neonato pré-termo de muito baixo peso, nascidos em hospitais públicos nas capitais da Região Nordeste.

## Método

Coorte prospectiva de base hospitalar dos nascidos vivos com idade gestacional de 23^0/7^ a 31^6/7^ semanas e peso ≥500g e <1.500g, nascidos em 19 maternidades públicas de referência nas capitais dos nove estados da Região Nordeste, de julho a dezembro de 2007. Foram excluídos os pacientes portadores de malformações congênitas maiores, aqueles transferidos de outras instituições e os que morreram em sala de parto. A pesquisa usou o banco de dados da Rede Norte-Nordeste de Saúde Perinatal (Renospe), uma iniciativa do Ministério da Saúde por intermédio da Área Técnica de Saúde da Criança. O projeto foi aprovado pelo Comitê de Ética em Pesquisa da Maternidade Escola Assis Chateaubriand e Unifesp e obtido o Termo de Fiel Depositário para uso dos dados da Renospe 2007.

A pesquisa desenvolvida pela Renospe, com dados coletados das Unidades de Terapia Intensiva Neonatal, avaliou 36 hospitais nos estados do Nordeste. No presente estudo, foram incluídos somente os hospitais localizados nas capitais de estado (29). Desses, foram excluídos dois por não terem maternidade e oito com número de pacientes inferior a 20 no período da coleta. Foram, portanto, incluídos 19 em nove capitais do Nordeste brasileiro.

Os hospitais selecionados atendiam exclusivamente pacientes do Sistema Único de Saúde. A coleta dos dados foi feita desde o momento da internação até a ocasião da alta ou óbito, a partir do prontuário do recém-nascido, de forma prospectiva por um pesquisador de campo treinado pela Renospe. Foram incluídas nessa coleta as características hospitalares, as características demográficas maternas e neonatais e a evolução clínica dos recém-nascidos avaliados.

Os 19 hospitais-maternidades foram avaliados por meio de formulário preenchido por profissionais de saúde, com relação aos recursos físicos, equipamentos, recursos humanos e iniciativas de busca de qualidade no atendimento, de acordo com metodologia publicada anteriormente,^9^ e foram propostas duas categorias: Nível 1 (N1) para as 13 maternidades com melhor infraestrutura e Nível 2 (N2) para as seis com infraestrutura menos qualificada.

Foram coletadas variáveis relacionadas às características demográficas maternas, intercorrências na gestação e uso do corticoide antenatal, considerado presente se pelo menos uma dose de corticoide foi aplicada antes do parto; e tipo de parto. Os dados neonatais avaliados relacionaram-se às características dos pacientes, aos procedimentos em sala de parto, e foi definido o uso de ventilação com pressão positiva (VPP) quando aplicada com balão e máscara ou cânula traqueal e a presença de reanimação avançada, se VPP com massagem cardíaca e/ou medicações. Foram coletadas informações relativas à temperatura do recém-nascido na admissão à UTI, considerada hipotermia <36°C,^10^ síndrome do desconforto respiratório (SDR), segundo critérios clínicos e radiológicos, e sepse precoce até 72 horas de vida com hemocultura positiva. Foram coletados dados relacionados a procedimentos e intervenções para o cuidado neonatal, como presença de transporte em incubadora entre a sala de parto e a UTI; ultrassonografia transfontanelar, se exame feito em qualquer momento da internação; uso de pressão positiva contínua nas vias aéreas (CPAP) em qualquer momento da internação; uso de ventilação pulmonar mecânica convencional em qualquer momento da internação; administração de surfactante, independentemente do momento usado; cateterismo umbilical, definido como introdução de cateter na artéria e/ou veia umbilical; uso de cateter central de inserção percutânea (PICC) em algum momento da internação; avaliação da dor em algum momento da internação, definida pela aplicação de qualquer escala de dor validada para o recém-nascido; uso de nutrição parenteral (NPP), quando essa foi usada em qualquer momento da internação e se foi anotado se seu início foi anterior a 24 horas de vida. O desfecho principal foi o óbito até 24 após o nascimento.

Na análise estatística, inicialmente compararam-se as variáveis de acordo com a presença ou ausência do desfecho por meio do teste *t* ou de Mann-Whitney para as variáveis quantitativas e por qui-quadrado ou teste exato de Fisher para as variáveis qualitativas. Para verificar os fatores associados ao óbito até 24 horas após o nascimento, efetuou-se a regressão logística com a estratégia passo a passo, com o método *backward.* Os fatores independentes introduzidos no modelo inicial foram todos aqueles que apresentaram valor de *p*<0,20 à análise univariada e se devia manter *p*<0,05 para permanecer no modelo. A associação entre as variáveis independentes e as variáveis-resposta foi expressa em razão de chances (OR) e intervalo de confiança de 95% (IC95%). O ajuste do modelo final foi avaliado pelo teste de Hosmer-Lemeshow. Em toda a análise estatística, o *software* usado foi o SPSS 17.0 (IBM SPSS Statistics for Windows, Version 17.0. Armonk, NY: IBM Corp) e foi significativo *p*<0,05.

## Resultados

No período do estudo ocorreram 27.991 nascimentos de recém-nascidos vivos nas 19 maternidades incluídas no estudo, dos quais 1.083 recém-nascidos com peso de 500-1499g, que corresponderam a 4% do total de nascimentos. Desses, foram excluídos do estudo 456 recém-nascidos e elegíveis 627 neonatos pré-termo com idade gestacional entre 23^0/7^ e 31^6/7^, peso entre 500 e 1.499g e sem malformações congênitas (fig. 1). Dos neonatos analisados, 76% nasceram nos 13 hospitais de Nível 1.

**Figura 1:** Recém-nascidos que compuseram a amostra do estudo com base no banco de dados da pesquisa da Rede Norte-Nordeste de Saúde Perinatal (Renospe) 2007.

Dentre os 627 recém-nascidos do estudo, 179 (29%) morreram antes de 168 horas de vida, dos quais 59 (33%) antes de 24 horas. Observa-se a seguinte distribuição dos óbitos de acordo com a idade gestacional: 216 pacientes com 23-27 semanas, dos quais 38 (18%) faleceram até 24 horas, e 411 neonatos com 28-31 semanas, dos quais 21 (5%) faleceram até 24 horas.

A distribuição das características maternas de acordo com a presença do óbito do recém-nascido até 24 horas de vida é mostrada na tabela 1. As características neonatais, as intervenções na sala de parto e as intercorrências clínicas nas primeiras 24 horas de vida, de acordo com a presença de óbito do recém-nascido no primeiro dia de vida, encontram-se na tabela 2. Na tabela 3 estão dispostas as variáveis relacionadas aos procedimentos e às intervenções para o cuidado neonatal, de acordo com a sobrevida ou óbito no primeiro dia de vida. Ressalta-se que dos 19 hospitais selecionados 13 (68%) situavam-se na categoria N1, detentores da melhor infraestrutura hospitalar, e, neles, a mortalidade no primeiro dia de vida dos 476 RNMBP atendidos foi de 7%, enquanto nos seis hospitais da categoria N2 e nos 151 pacientes assistidos esse número foi de 17%.

**Tabela 1:** Variáveis maternas, de acordo com a presença de óbito neonatal com 24 horas após o nascimento de neonatos pré‐termo de muito baixo peso nas capitais da região Nordeste

	Óbitos <24h (n=59)	Vivos ≥24h (n=568)	*p* ‐valor
Idade materna (anos) a	24±7	25±7	0,313
Idade <20 anos	19 (32%)	140 (25%)	0,134
Escolaridade <8 anos	32 (54%)	272 (48%)	0,214
Sem pré‐natal	19 (35%)	88 (15%)	**0,003**
Gestação múltipla	9 (15%)	78 (14%)	0,436
Diabetes na gestação	0	8 (2%)	0,500
Síndrome hipertensiva	7 (15%)	178 (34%)	**0,004**
Infecção periparto	13 (25%)	186 (36%)	0,205
Corticoide antenatal	15 (27%)	275 (51%)	**0,001**
Parto cesárea	16 (28%)	270 (48%)	**0,003**

a Variável descrita em média±desvio‐padrão.

**Tabela 2:** Características neonatais, de acordo com a presença de óbito neonatal com 24 após o nascimento de neonatos pré‐termo de muito baixo peso nas capitais da região Nordeste

	Óbitos <24h (n=59)	Vivos ≥24h (n=568)	*p* ‐valor
PN <1000g	45 (76%)	242 (43%)	**<0,001**
Peso ao nascer (g) a	854±235	1040±252	**<0,001**
IG <28 semanas	38 (64%)	178 (31%)	**<0,001**
IG (semanas) a	27±2	28±2	**<0,001**
Sexo masculino	40 (68%)	278 (49%)	**<0,001**
Apgar 1 minuto <3	25 (45%)	57 (10%)	**<0,001**
Apgar 5 minutos <7	34 (61%)	81 (15%)	**<0,001**
Uso de VPP na SP	46 (82%)	338 (60%)	**<0,001**
Reanimação avançada	11 (21%)	26 (5%)	**<0,001**
Temp. à admissão (°C) a	35,3±0,8	35,6±0,7	**0,003**
Hipotermia à admissão	41 (91%)	429 (82%)	**0,008**
SDR	51 (90%)	487 (87%)	0,360
Sepse precoce HC+	2 (4%)	32 (6%)	0,436

a Variável descrita em média±desvio‐padrão; PN, peso ao nascer; IG, idade gestacional; VPP, ventilação com pressão positiva; SP, sala de parto; reanimação avançada, VPP com massagem cardíaca e/ou medicações; SDR, síndrome de desconforto respiratório; HC+, hemocultura positiva.

**Tabela 3:** Procedimentos e intervenções para o cuidado neonatal diagnóstico e terapêutico, de acordo com a presença de óbito neonatal com 24 após o nascimento de neonatos pré‐termo de muito baixo peso nas capitais da região Nordeste

	Óbitos <24h (n=59)	Vivos ≥24h (n=568)	*p* ‐valor
Transporte em incubadora a	14 (26%)	248 (45%)	**0,004**
Uso de surfactante	35 (59%)	366 (64%)	0,260
Surfactante até 2 horas de vida	23 (66%)	243 (66%)	0,533
CPAP	11 (19%)	423 (75%)	**<0,001**
Ventilação mecânica	51 (86%)	418 (74%)	**0,018**
US transfontanelar	1 (2%)	316 (56%)	**<0,001**
Cateterismo umbilical	43 (73%)	436 (77%)	0 **,** 300
PICC	1 (2%)	133 (23%)	**<0,001**
Uso escala de dor b	2 (4%)	152 (28%)	**<0,001**
Nutrição parenteral	5 (9%)	415 (73%)	**<0,001**
NPP <24h [n NPP<24h /n NPP (%)]	2/5 (40%)	192/420 (46%)	0 **,** 571
Categoria hospitalar (N1) c	33 (56%)	443 (78%)	**<0001**

a Transporte do Centro Obstétrico à UTI ao nascimento; HV, horas de vida; CPAP, pressão de distensão contínua em vias aéreas por prongas nasais; US, ultrassom; PICC, cateter venoso central de inserção percutânea.

b Uso de qualquer escala validada de avaliação da dor; NPP, nutrição parenteral; NPP24, início da alimentação parenteral nas primeiras 24 horas de vida.

c Categoria hospitalar N1 quando o hospital atendeu mais de 60% dos itens descritos no método.

A tabela 4 mostra o resultado da análise multivariada para o desfecho óbito no primeiro dia de vida. Observa-se que a melhor infraestrutura hospitalar, ou seja, hospitais categorizados como Nível 1, foi fator de proteção para o desfecho analisado, o peso ao nascer <1.000g, o Boletim de Apgar 5° minuto <7 e o sexo masculino se comportaram como fatores de risco. O modelo de regressão logística foi ajustado para as variáveis presença de pré-natal, parto cesárea e uso de corticoide antenatal. O teste de Hosmer-Lemeshow mostra ajuste adequado do modelo, com *p*=0,712.

**Tabela 4:** Variáveis independentes associadas à variável‐resposta “óbito até 24 horas após o nascimento de neonatos pré‐termo de muito baixo peso nas capitais da região Nordeste”: modelo final da análise de regressão logística

	*Odds Ratio*	Intervalo de confiança de 96%
Categoria hospitalar N1	0,34	0,17‐0,71
Peso ao nascer <1.000g	2,94	1,32‐6,53
Apgar 5 minutos <7	7,17	3,46‐14,88
Sexo masculino	2,99	1,39‐6,47

Modelo foi ajustado para presença de pré‐natal, parto cesárea e uso de corticoide antenatal.

## Discussão

Este estudo multicêntrico revela que a mortalidade neonatal nas primeiras 24 horas de vida é elevada nos recém-nascidos pré-termo de muito baixo peso nas capitais da região Nordeste, se comparada com a de regiões mais desenvolvidas do Brasil e com países desenvolvidos: dos 627 neonatos estudados, 59 (9,4%) morreram nas primeiras 24 horas. No estudo da Rede Brasileira de Pesquisas Neonatais, em 2004, com maternidades públicas universitárias do Sul e Sudeste, dos 560 neonatos avaliados, excluídos os óbitos em sala de parto, 25 (4,5%) morreram nas primeiras 24 horas. Salienta-se que foram incluídos no estudo citado pacientes com peso entre 400 e 1.499g, o que pode ter contribuído para elevar tal percentual.^11^ Mohamed et al.,^12^ em estudo de coorte entre 1997 e 2004 nos Estados Unidos em neonatos com peso ao nascer entre 500-1.499g, observaram que dentre os 91.578 estudados 4.579 (5%) morreram nas primeiras 24 horas. Quanto mais precoce o óbito do recém-nascido, mais ligado está às condições antenatais, ao periparto e ao parto propriamente dito. Destaca-se, para as mortes no primeiro dia após o nascimento, a sua ligação com determinantes sociais e econômicos relacionados à qualidade de atenção à saúde da gestante.^13^ Foi nesse contexto que se buscou entender como se comportavam os fatores associados ao óbito com 24 horas após o nascimento.

Dentre os fatores associados ao óbito neonatal no primeiro dia de vida, ressaltam-se três grupos de variáveis: as ligadas à qualidade do atendimento perinatal (categoria hospitalar), aquelas relacionadas às variáveis biológicas neonatais (peso ao nascer e sexo masculino) e as que refletem o atendimento ao trabalho de parto e ao parto (Apgar de 5 minutos).

No que diz respeito à estrutura hospitalar, todos os hospitais do estudo pertenciam ao Sistema Único de Saúde (SUS) e vários autores indicam taxas de mortalidade neonatal elevadas em hospitais públicos do SUS.^6^^,^ 13 ^,^ 14 É preciso lembrar que as características socioeconômicas da população usuária do SUS, por si, podem acarretar piores resultados de sobrevida. Entretanto, Alleman et al.^15^ apontam que as diferenças de mortalidade neonatal entre as diversas unidades não podem ser explicadas somente pela diversidade das características dos pacientes aí admitidos, mas devem estar mais relacionadas ao uso de intervenções preditoras de sobrevida. Nesse sentido, observa-se que a melhor infraestrutura hospitalar, no presente estudo, protegeu o recém-nascido em relação ao óbito até 24 horas após o nascimento, diminuiu a chance de sua ocorrência em 66%. Ressalta-se que, na ausência de um modelo padronizado de avaliação de maternidades associado ao risco de morte neonatal, foi estabelecido um escore ponderado das características relacionadas à estrutura hospitalar, no qual as características menos frequentes foram mais valorizadas. De acordo com esse escore, observou-se que a mortalidade no primeiro dia de vida dos RNMBP foi mais de duas vezes maior nos hospitais com pior infraestrutura (N2) comparados com os que contavam com melhores condições (N1). Dentre as características estudadas, recursos tecnológicos mais sofisticados, como ultrassonografia e ecocardiografia no leito, e a possibilidade de refletir sobre a prática médica empregada e aprender com possíveis erros e omissões por meio de reuniões clínicas estavam ausentes da maioria das instituições classificadas como N2. Ou seja, embora todas as maternidades analisadas fossem públicas e ligadas ao SUS, algumas investiram mais em recursos diagnósticos para pacientes prematuros, dependentes de tecnologia para sua sobrevivência, e algumas investiram mais na capacitação de recursos humanos, fundamentais para aplicar os recursos tecnológicos nessa população de recém-nascidos extremamente vulneráveis.

No que concerne aos fatores biológicos ligados ao óbito neonatal até 24 horas após o nascimento, destacam-se o extremo baixo peso ao nascer e o sexo masculino. Dentre os 287 recém-nascidos <1.000g analisados no presente estudo, 45 (15,7%) morreram até 24 horas, perfizeram 76% dos neonatos que morreram nesse período. O peso ao nascer <1.000g aumentou em três vezes o risco de morte nas primeiras 24 horas, comparado com o peso de 1.000-1.499g. Na rede americana Neonatal Network, dentre os 6.780 neonatos pré-termo de extremo baixo peso nascidos entre 1998 e 2003, a mortalidade foi de 14,3% nas primeiras 24 horas.^16^ Em coorte de base populacional em São Paulo entre 2000 e 2001, dos óbitos ocorridos até 12 horas de vida, 86% tinham peso ao nascer <1.000g.^17^ Lee et al.^18^ propuseram um modelo que inclui tanto a idade gestacional como o peso de nascimento para analisar o risco de óbito, com elevação de tal risco a cada decréscimo de 100g no peso de nascimento e de uma semana na idade gestacional. No presente estudo, nos 109 RNMBP entre 500-749g, a mortalidade nas primeiras 24 horas foi de 19,3%, enquanto nos 178 com 750-999g esse valor foi de 13,5%. Mohamed et al.^12^ analisaram a sobrevida de RNMBP entre 1997 e 2004 nos Estados Unidos. Nos 18.863 neonatos com peso entre 500-749g, a mortalidade no primeiro dia de vida foi de 19,6%, enquanto nos 17.433 com peso entre 750-999g foi de 3,1%. Provavelmente, a menor mortalidade na faixa de peso ao nascer entre 500-749g das maternidades nordestinas comparada com as americanas reflete um número grande de recém-nascidos com restrição de crescimento intrauterino e idade gestacional mais avançada, o que favorece a chance de sobrevida. De fato, dos 109 RN com peso entre 500-749g, 26 (24%) tinham idade gestacional de 28-31 semanas. Ressalta-se ainda que, no presente estudo, dentre os 627 neonatos com idade gestacional de 23 semanas a 31 semanas e seis dias, peso ao nascer entre 500 e 1499g e sem malformações, 21 (3%) morreram na sala de parto e perderam, assim, a oportunidade da assistência na Unidade de Terapia Intensiva.

O sexo masculino apresentou forte associação com o óbito nas primeiras 24 horas de vida, com risco três vezes maior de morte comparado com o feminino. O mesmo achado foi evidenciado na mortalidade intra-hospitalar em outros estudos. 19 ^,^ 20 Itabashi et al.^21^ avaliaram 3.065 recém-nascidos japoneses de extremo baixo peso e observaram risco 1,6 maior de morte hospitalar no sexo masculino. Achados de pesquisa australiana apontam que o crescimento e a sobrevida fetal são mediados por funções sexo-específicas da placenta humana, com melhor adaptação e resposta ao estresse oxidativo no sexo feminino.^22^

Dentre os fatores relacionados à atenção ao parto que se associaram à mortalidade nas primeiras 24 horas após o nascimento, destaca-se o Escore de Apgar com 5 minutos <7. Em uma investigação de base populacional nos Estados Unidos, entre 2001-2002, Lee et al.^18^ avaliaram uma coorte de 690.933 neonatos entre 24-36 semanas de idade gestacional e observaram associação de baixos escores de Apgar no 5° minuto de vida com mortalidade neonatal. Na Suécia, entre 2000 e 2002, a análise de 156 neonatos pré-termo com 23 e 24 semanas de idade gestacional identificou também associação de baixos escores de Apgar no 5° minuto com a mortalidade neonatal nas primeiras 24 horas de vida.^23^ O correlato da baixa vitalidade ao nascer é a necessidade de procedimentos avançados de reanimação neonatal, definidos como intubação e/ou massagem cardíaca e/ou uso de medicações em sala de parto. Estudo da Rede Americana de Pesquisas Neonatais, com dados de 9.565 neonatos pré-termo entre 22-28 semanas, mostra que 67% receberam ventilação ao nascer por meio de cânula traqueal, 8% necessitaram de massagem cardíaca e 5% de medicações.^24^ Dados nacionais indicam que a necessidade de reanimação avançada em neonatos pré-termo de muito baixo peso aumenta em duas vezes a chance de óbito intra-hospitalar ou de sobrevida com displasia broncopulmonar e/ou hemorragia intracraniana grave.^25^ No presente estudo, observou-se forte associação do Boletim de Apgar no 5° minuto <7 com o óbito nas primeiras 24 horas de vida. Tal risco foi 7,2 maior do que o daqueles com Apgar no 5° minuto de 8-10. A adequada atenção ao parto dos recém-nascidos pré-termo nascidos nas maternidades de referência das capitais nordestinas delineia-se como medida prioritária para atenuar as dificuldades de transição para a vida extrauterina, facilitar a adaptação cardiorrespiratória e possibilitar estabilidade clínica e redução da mortalidade no primeiro dia de vida.

É importante enfatizar que o uso de dados secundários apresenta limitações e dificuldades inerentes à própria metodologia e favorece falhas no preenchimento dos formulários. Assim, oito hospitais foram excluídos da pesquisa por apresentar amostra não representativa de suas instituições. Além do mais, o lapso de sete anos entre coleta de dados e sua análise mostra as dificuldades para a consolidação de informações em unidades em que não há capacitação para a pesquisa. Apesar disso, trata-se do primeiro estudo com coleta prospectiva de dados de maternidades de referência da Região Nordeste que permite delinear um retrato do atendimento ao nascimento e parto de neonatos pré-termo de muito baixo peso, que contribuem substancialmente para a mortalidade neonatal e infantil e influenciam o índice de desenvolvimento humano desses locais. Nesse contexto, os resultados permitem concluir que a mortalidade neonatal precoce especialmente nas primeiras 24 horas é elevada nos pacientes pré-termo de muito baixo peso nas capitais da Região Nordeste se comparada com a de regiões mais desenvolvidas do Brasil e a de países desenvolvidos. Os achados revelam a existência de falhas nas unidades de terapia intensiva avaliadas, com infraestrutura hospitalar deficiente e elevada contribuição do extremo baixo peso e da asfixia perinatal para a mortalidade nas primeiras 24 horas de vida. A superação desses dados requer uma política governamental que, de forma clara, atribua responsabilidades para ações direcionadas à melhoria de qualidade, com definições de prioridades, seleção e definições de intervenções e avaliação constantes.
